# The miR-210 Primed Endothelial Progenitor Cell Exosomes Alleviate Acute Ischemic Brain Injury

**DOI:** 10.2174/011574888X266357230923113642

**Published:** 2023-11-10

**Authors:** Jinju Wang, Shuzhen Chen, Harshal Sawant, Yanfang Chen, Ji Chen Bihl

**Affiliations:** 1 Department of Biomedical Sciences, Joan C Edwards School of Medicine, Marshall University, Huntington, WV25755, USA;; 2 Department of Pharmacology and Toxicology, Boonshoft School of Medicine, Wright State University, Dayton, OH45435, USA

**Keywords:** Exosomes, endothelial progenitor cells (EPCs), miR-210, acute ischemic stroke, oxidative stress, cell apoptosis

## Abstract

**Background:**

Stem cell-released exosomes (EXs) have shown beneficial effects on regenerative diseases. Our previous study has revealed that EXs of endothelial progenitor cells (EPC-EXs) can elicit favorable effects on endothelial function. EXs may vary greatly in size, composition, and cargo uptake rate depending on the origins and stimulus; notably, EXs are promising vehicles for delivering microRNAs (miRs). Since miR-210 is known to protect cerebral endothelial cell mitochondria by reducing oxidative stress, here we study the effects of miR-210-loaded EPC-EXs (miR210-EPC-EXs) on ischemic brain damage in acute ischemic stroke (IS).

**Methods:**

The miR210-EPC-EXs were generated from EPCs transfected with miR-210 mimic. Middle cerebral artery occlusion (MCAO) surgery was performed to induce acute IS in C57BL/6 mice. EPC-EXs or miR210-EPC-EXs were administrated *via* tail vein injection 2 hrs after IS. To explore the potential mechanisms, inhibitors of the vascular endothelial growth factor receptor 2 (VEGFR2)/PI3 kinase (PI3K) or tyrosine receptor kinase B (TrkB)/PI3k pathways were used. The brain tissue was collected after treatments for infarct size, cell apoptosis, oxidative stress, and protein expression (VEGFR2, TrkB) analyses on day two. The neurological deficit score (NDS) was evaluated before collecting the samples.

**Results:**

1) As compared to EPC-EXs, miR210-EPC-EXs profoundly reduced the infarct volume and improved the NDS on day two post-IS. 2) Fewer apoptosis cells were detected in the peri-infarct brain of mice treated with miR210-EPC-EXs than in EPC-EXs-treated mice. Meanwhile, the oxidative stress was profoundly reduced by miR210-EPC-EXs. 3) The ratios of p-PI3k/PI3k, p-VEGFR2/VEGFR2, and p-TrkB/TrkB in the ipsilateral brain were raised by miR210-EPC-EXs treatment. These effects could be significantly blocked or partially inhibited by PI3k, VEGFR2, or TrkB pathway inhibitors.

**Conclusion:**

These findings suggest that miR210-EPC-EXs protect the brain from acute ischemia-induced cell apoptosis and oxidative stress partially through the VEGFR2/PI3k and TrkB/PI3k signal pathways.

## 1 INTRODUCTION

Ischemic stroke (IS) remains one of the most severe health problems worldwide. For treating acute IS, thrombolytic therapy is limited by a four-hour therapeutic time window. Interventional therapies, such as angioplasty and stenting, have a high risk of re-stroke within the first year [1]. Increasing evidence suggests that stem cell-based therapy could be a promising strategy for treating IS [2]. Liao *et al*. have revealed that EPCs can protect the neurons from ischemic injury by repairing secretory functions and vascular endothelium [3]. Our previous studies have demonstrated that transfusion of endothelial progenitor cells (EPCs) protects the brain from acute ischemic injury *via* cerebrovascular protection in a mouse IS model [4, 5].

Exosomes (EXs) are a type of extracellular vesicle that has been recognized as a novel way of cell-cell communication by transferring their carried information, such as mRNAs and microRNAs (miRs) [6, 7]. EXs are also considered an important player in the tissue “microenvironment” [8]. Increasing studies suggest that the benefits of stem cells such as EPCs are ascribed to their released EXs [9]. Indeed, our recent study reveals that EXs from EPCs (EPC-EXs) can merge with brain cells and protect the brain against ischemia-induced cell apoptosis, preserving cerebral blood flow in a diabetic stroke mouse model [10]. Among the molecules that EXs carry, miRs show the most important role in regulating cellular function [11, 12]. The function of EXs is highly related to their carried molecules. Exosomal miRs are one of the major executors of EXs *via* conveying the benefit of one cell to another cell [13]. For instance, the exosomal miR-126 has been shown to contribute to the therapeutic effects of EPC-EXs in IS in a diabetic mouse stroke model [10]. The miR-126 is a positive regulator of angiogenesis for endothelial cells (ECs) [14]. Overexpression of miR-126 in ECs could enhance vascular endothelial growth factor A (VEGF-A) activity, which activates the PI3 kinase (PI3K) signaling to suppress cell apoptosis [15, 16].

It is known that miR-210 is a master hypoxamir [17]. It is reported that miR-210 is involved in cell protection with anti-apoptotic and anti-oxidant properties by reducing mitochondrial reactive oxygen species (ROS) production [18-20]. Our group has found that miR-210 could enhance the protective effects of EPC-EXs on protecting ECs and neurons against hypoxia-reoxygenation injury [21, 22], as well as boost the favorable effects of neural progenitor cells derived EXs on angiotensin-II injured ECs [23]. However, clinical studies found that the miR-210 level is significantly decreased in stroke patients at 7 and 14 days after stroke onset and that the circulating miR-210 level is positively related to the outcome of stroke patients [24]. Zeng *et al*. found that the overexpression of miR-210 could improve the long-term outcomes after focal cerebral ischemia in mice [25]. All these findings suggest that miR-210 might be a therapeutic target for treating IS.

Increasing studies indicate that the functions of EXs vary based on the origin of cells, their cellular status, and their cargo. EPC-EXs released under starvation or inflammation stimulation function differently on hypoxia/reoxygenation-injured endothelial cells, which is related to their carried miRs, such as miR-126 [26]. We have recently revealed that exercise intervention can modulate the release and function of circulating EPC-EXs by affecting the package of miR-126 [27, 28]. Some research groups utilize EXs as drug carriers or biomaterials, providing a platform to enhance EX bioavailability and maximize EX regenerative capacity *in vivo* [29, 30]. In the present study, we aimed to investigate whether miR-210 priming EPC-EXs (miR210-EPC-EXs) could exhibit enhanced effects on treating acute IS by combining the beneficial effects of EPC-EX (miR-126) and miR-210.

## 2 MATERIALS AND METHODS

### 2.1 Preparation of miR-210-EPC-EXs

The EPCs (Celprogen, Torrance, CA) were cultured in EPC complete growth medium with serum and antibiotics in an incubator with 5% CO_2_ at 37°C as previously published [21, 31]. To generate miR210-EPC-EXs, EPCs were transfected with miR-210 mimics (1 nmol/L, Qiagen) using Dharmafect 1 transfection reagent (Dharmacon) for 48 hours in EPC complete growth medium, followed by another 48-hour serum-free medium culture. The culture medium was then collected for EX isolation, as we reported [31, 32]. EXs released from non-transfected EPCs cultured in serum-free media served as control. The miR210-EPC-EXs and EPC-EXs pellet were suspended with 100μl sterile-filtered phosphate buffer saline (PBS) for Nanotracking Analysis (NTA) or intravenous injection.

### 2.2 Numeration of EPC-EXs by NTA

The concentration and size distribution of EXs were measured by the NS300 instrument (Nanosight, Amesbury, UK) prior to administration into the experimental mice, as we previously described [26].

### 2.3 Animals

C57BL/6 J mice (Male and Female (50%/50%), 10-12 weeks old) purchased from the Jackson Laboratory (Bar Harbor, ME, USA) were used in this study. All mice were maintained in a 22°C room with a 12 h light/dark cycle and fed with standard chow and drinking water ad libitum. Body weights were recorded weekly. The ovariectomy was not performed upfront. All experimental procedures were approved by the Marshall University Laboratory Animal Care and Use Committee (LACUC) and followed the Guide for the Care and Use of Laboratory Animals issued by the National Institutes of Health.

### 2.4 Middle Cerebral artery Occlusion (MCAO) Surgery and Neurological Deficit Score (NDS) Evaluation

As we previously reported, the MCAO surgery was conducted by inserting an intraluminal filament [4, 5]. Briefly, mice were anesthetized by isoflurane (3-5% for induction, 2-2.5% isoflurane for maintenance), and the animal's body temperature was maintained using a water-jacketed heating pad. The left common carotid artery was exposed and ligated distal to the bifurcation. The left external carotid artery was ligated and cut to expose the left internal carotid artery. A suture was placed under the internal carotid artery and lightly lifted to prevent blood backflow from the head. Then, a small incision was made on the common carotid artery between the ligation and carotid bifurcation. A 7-0 nylon monofilament suture with a rounded head coated with poly-l-lysine was inserted through the small incision and advanced into the internal carotid artery until resistance was detected (about 10 mm distal to the bifurcation). The suture was left in place with ligation for permanent MCAO. Pain and discomfort were minimized by injection of Buprenorphine (3.25 mg/kg body weight, s.c) after the operation. The neurological function of mice was evaluated by using the 5- point scale one day before the surgery and two days after the treatment. The five points are 0, normal motor function; 1, flexion of the contralateral torso and forelimb upon lifting the whole animal by the tail; 2, circling to the contralateral side but normal posture at rest; 3, leaning to the contralateral side at rest; 4, no spontaneous motor activity. The investigator who scored the neurologic behavior was blind to the animal grouping information.

### 2.5 Experimental Groups

Two hours after MCAO surgery, mice were randomly divided into six experimental groups: vehicle, EPC-EXs, miR-210-EPC-EXs, miR-210-EPC-EXs + ly294002, miR-210-EPC-EXs + su1498, miR-210-EPC-EXs + k252a. Twelve mice were used for neurological deficit score analysis in each group, and eight were used for each postmortem analysis. The sample size was determined by following previous publications. The miR-210-EPC-EXs or EPC-EXs (1×10^11^ particles) resuspended with 100ul sterile-filtered PBS were intravenously injected *via* the caudal vein, as we reported [10]. The pathway inhibitors ly294002 (PI3k inhibitor; 10 mg/kg, i.p., Cayman Chemical), su1498 (VEGFR2 pathway inhibitor; 9 mg/kg, i.p, BioVision), and k252a [tyrosine receptor kinase B (TrkB) pathway inhibitor; 10 μg/kg/d, i.p, BioVision] were used per previous reports [33-35]. The pathway inhibitors were used to investigate if the underlying mechanisms of the protective effects of miR-210 are related to the VEGFR2/PI3k and TrkB/PI3k pathways. The brain samples were collected on day 2 to study the acute injury of IS after treatment for different measurements.

### 2.6 Brain Sample Collection and Preparation

Mice were euthanized 48 hrs after treatment. The animals were transcardially perfused with PBS and 4% paraformaldehyde (PFA) in PBS, pH 7.4. The brains were immediately collected and fixed in 4% PFA overnight and 4% PFA plus 30% sucrose for three days. The brains were then cut into coronal sections (20 μm) and sequentially put into six separate wells of a six-well plate containing 2 ml of PBS. Five serial sections spaced 300 um apart were selected for staining to represent one brain. Frozen sections were used with a cryostat (Leica).

### 2.7 Cerebral Infarct Volume Assesement

The cerebral injury was assessed by 2,3,5-Triphenyltetrazolium chloride (TTC) staining. TTC staining was conducted to evaluate the infarct volume of the brain on day two post-MCAO. Images of all stained slices were captured using a flatbed scanner. The area of infarction was measured by using Image J software.

### 2.8 *In situ* Apoptosis Detection in the Brain Tissue

The cellular apoptosis in the brain tissue was measured by terminal deoxynucleotidyl transferase (TdT) dUTP Nick-End Labeling (TUNEL) assay kit (Roche, Switzerland) according to the manufacturer's instructions. In brief, brain slices (20 μm) were mounted on gelatin-coated slides and permeabilized with 0.1% TritonX-100/0.1% sodium citrate for 2 minutes. And then, the slides were washed and incubated with a freshly prepared TUNEL reaction mixture in an incubator for 60 minutes at 37°C in the dark. Cell nuclei were stained with 4’, 6-diamidino-2 -phenylindole (DAPI, 1 μg/mL; Wako Pure Chemical Industries Ltd). Tissue samples were examined under a fluorescence microscope (Nikon, Eclipse E600). An average of five brain sections spaced 300 um apart, from rostral to caudal, were selected to represent one mouse. Randomly five microscopic areas were counted in the peri-infarct area for every brain section. The peri-infarct area was defined as the area approximately 200 um lateral from the infarct border by following the previous publications [36, 37]. The apoptotic cell was determined as the number of TUNEL-positive cells per field.

### 2.9 Intracellular ROS Generation in Brain Tissue

Dihydroethidium (DHE, Sigma-Aldrich) staining was applied to detect the intracellular ROS generation in the peri-infarct brain tissue [21, 23]. DHE is a superoxide indicator that, when oxidized primarily by superoxide, results in 2-hydroxy ethidium. The brain slices (20-um thickness) were probed with 50 μM DHE solution (in the dark) for 30 minutes at 37°C. The images were taken under a fluorescence microscope (Nikon, Eclipse E600). An average of five brain sections, from rostral to caudal, were selected to represent one mouse. Randomly five microscopic areas were counted for every brain slide. The fluorescence intensity of DHE was analyzed using Image J (NIH).

### 2.10 MiR Level Measurement in the Brain

After the treatment, the levels of miR-210 and miR-126 in the ipsilateral brain tissue were determined by Real-time PCR, as we previously published [21, 23]. Briefly, the total RNAs were extracted from the brain tissue using the TRIzol reagent, and the RNA concentration was measured using NanoDrop 2000 Spectrophotometer (Thermo Fisher Scientific). cDNA was synthesized using the PrimeScript RT reagent kit (Takara Bio Inc.) following the manufacturer’s instructions. qRT-PCR was carried out using miR-210 and miR-126 specific primers and SYBR Premix Ex Taq kit (Takara Bio Inc.) on a real-time PCR instrument (Bio-Rad). RNA U6 was used as an internal control. The primers of miR-210: RT primer: 5’- GTC GTA TCC AGT GCA GGG TCC GAG GTA TTC GCA CTG GATACG AC GAC TGT-3’; Forward primer: 5’- CAC GCA GTC GTA TCC AGT GCA GG-3’; Reverse primer: 5′-CCA GTG CAG GGT CCG AGG TA-3′. The primers of miR-126: RT primer: 5’- GTC GTA TCC AGT GCA GGG TCC GAG GTA TTC GCA CTG GATACG AC CGC ATT -3’; Forward primer: 5’- AGG CGC TCG TAC CGT GAG TAA TA - 3’; Reverse primer: 5′-CCA GTG CAG GGT CCG AGG TA-3′. The primers of U6: 5’-CTCGCTTCGGCAGCACA-3’ (forward); 5’-AACGCTTCACGAATTTGCGT-3’ (reverse). The expressions of miR-210 and miR-126 were normalized to U6 and calculated using the 2−ΔΔCT method.

### 2.11 Analyses of Angiogenic and Neurotrophic Factors in the Brain

The proteins were extracted from the brain tissue and quantified by a BCA protein assay. The levels of VEGF and brain-derived neurotrophic factor (BDNF) in the tissue samples were measured by using ELISA kits (VEGF: Abcam; BDNF: MyBioSource) according to the manufacturer's instructions. The data were normalized and expressed as a fold of the level in the vehicle group.

### 2.12 Western Blot Analysis

The proteins of brain tissues (stroke area) in different treatment groups were extracted with cell lysis buffer (Thermo Fisher Scientific). The protein lysates (35 μg) were electrophoresed, transferred onto PVDF membranes, and incubated with primary antibody against PI3k (1:100; Invitrogen), p-PI3k (1:500; Invitrogen), p-VEGFR2 (1:1000; Abcam), VEGFR2 (1:1000; Abcam), p-TrkB (1:1000; Abcam), TrkB (1:1000; Abcam), β-actin (1:4000; Sigma) at 4°C overnight. Then all membranes were washed and incubated with horseradish-peroxidase-conjugated anti-rabbit or anti- mouse IgG (1:40000; Jackson Immuno Research Lab) for 2 hr at room temperature. Blots were developed with enhanced chemiluminescence developing solutions, and images were quantified under ImageJ software.

### 2.13 Statistical Analysis

The neurological deficit scores were expressed as median (range). The neurological deficit scores among different groups were compared by a Kruskal-Wallis test. When the Kruskal-Wallis test showed a significant difference, the Mann-Whitney U tests were applied as a post hoc test. All other data are presented as mean ± SD. Two group comparison was analyzed by student t-test. Multiple comparisons were analyzed by two-way ANOVA (SPSS version 16.0; SPSS, Chicago, IL, USA) followed by the Tukey test. For all tests, a P-value <0.05 was considered significant.

## 3 RESULTS

### 3.1 The levels of miR-210 and miR-126, as Well as VEGF and BDNF Levels, were Up-regulated by miR210-EPC-EXs in the Ipsilateral Brain of C57BL/6 J Mice on Day 2 after IS Onset

To determine whether the miR210-EPC-EX infusion could affect the levels of miR-210 and miR-126 in the brain tissue, we extracted the total RNAs from the ipsilateral brain samples and analyzed the miR level. As shown in Figs. (**1A**, **B**), EPC-EX or miR210-EPC-EX infusion raised the miR-126 level in the ipsilateral brain compared to that in the vehicle group (*p* < 0.05, *vs.* vehicle). The miR-210 level was increased by miR210-EPC-EX infusion (*p* < 0.05, *vs.* vehicle or EPC-EXs), while no significant difference in miR-210 expression was observed between the vehicle and EPC-EX infusion group (p>0.05).

**Fig. (1):**
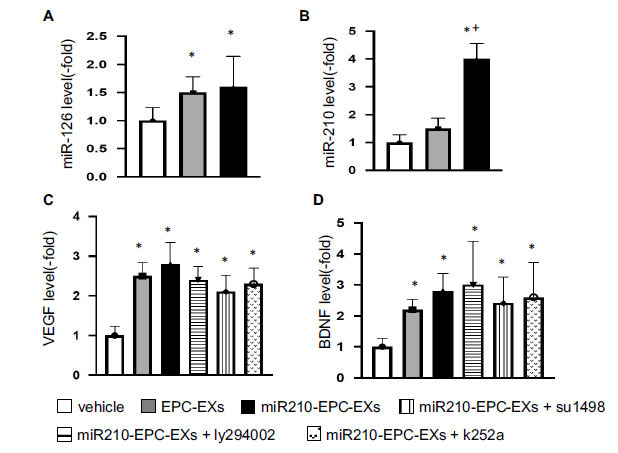
The levels of miR-126, miR-210, VEGF, and BDNF in the ipsilateral brain tissue on day 2 following MCAO. (**A**, **B**) Summarized data for miR-126 and miR-210 levels in the ipsilateral brain tissue. (**C**-**D**), summarized data on VEGF and BDNF levels in the brain tissue in different treatment groups. *p < 0.05, *vs.* vehicle; ^+^*p* < 0.05, *vs.* EPC-EXs. n = 8 per group.

Regarding the growth factor levels, we focused on VEGF and BDNF in the brain tissue. As revealed by the ELISA assay (Figs. **1C**, **D**), the levels of VEGF and BDNF were also increased by EPC-EX or miR210-EPC-EX infusion (*p* < 0.05, *vs.* vehicle).

### 3.2 miR210-EPC-EXs Significantly Reduced the Infarct Volume and Improved the Neurological Deficit Score in C57BL/6 J Mice on Day 2 after IS Onset

As previously reported [5], the brain tissue was collected and stained with TTC two days after the treatment. As shown in Figs. (**2A**, **B**), the white area indicates cerebral infarcted tissue. As compared to the vehicle group (no treatment; PBS only), EPC-EXs infusion decreased the infarct size (by ~ 21.4%), which was further reduced by the miR210-EPC-EXs treatment (by ~ 53.5%). Pre-treatment with PI3k inhibitor ly294002 significantly blocked the effects induced by miR210-EPC-EXs, while VEGFRs inhibitor su1498 and TrkB inhibitor k252a partially blocked the effects. Together these data suggest that the protective effects exhibited by miR210-EPC-EXs were modulated by the VEGFR2/PI3k and TrkB/PI3k signal pathways.

To determine the neurological function, we conducted the neurological deficit score analysis. Our data showed that EPC-EXs treatment significantly alleviated the neurological deficit in the C57BL/6 J mice on day 2 after IS onset (*p* < 0.05, *vs.* vehicle), which was further profoundly improved by miR210-EPC-EXs (*p* < 0.05, *vs.* EPC-EXs). PI3k inhibitor ly294002 diminished the improved neurological deficit function elicited by miR210-EPC-EXs (*p* < 0.05, *vs.* miR210-EPC-EXs), while su1498 (*p* < 0.05, *vs.* miR210-EPC-EXs) and k252a (*p* < 0.05, *vs.* miR210-EPC-EXs) partially reduced the effects, indicating that the VEGFR2/PI3k and TrkB/PI3k signal pathways are responsible for the protective effects of miR210-EPC-EXs.

### 3.3 miR210-EPC-EXs Remarkably Alleviated Cell Apoptosis in the Peri-infarct Brain Tissue of C57BL/6 J Mice on Day 2 after IS Onset

Cell loss in the ipsilateral brain in the acute IS stage was assessed by Tunnel staining. As shown in Fig. (**3**), there were a greater number of Tunnel+ cells (168 ± 15 Tunnel+ cells/field) in the ipsilateral cerebral tissue in the vehicle mice. EPC-EXs treatment significantly reduced the number of Tunnel+ cells (135 ± 10 Tunnel+ cells/field, *p* < 0.05 *vs.* vehicle) in the ipsilateral brain, and miR210-EPC-EXs further enhanced the anti-apoptotic effect of EPC-EXs (72 ± 6 Tunnel+ cells/field, *p* < 0.05 *vs.* EPC-EXs). Blocking the PI3k pathway with the pathway-specific inhibitor ly294002 abolished (164 ± 14 Tunnel+ cells/field, *p* < 0.05 *vs.* miR210-EPC-EXs), whereas VEGFR2 or TrkB inhibitors partially blocked the anti-apoptotic role of miR210-EPC-EXs in C57BL/6 J mice in the acute phase of IS as revealed by a decreased number of Tunnel+ cells (86 ± 7 Tunnel+ cells/field for su1458, and 89 ± 8 Tunnel+ cells/field for k252a, *p* < 0.05 *vs.* vehicle or EPC-EXs).

### 3.4 miR210-EPC-EXs Alleviated Oxidative Stress in the Peri-infarct Area of C57BL/6 J Mice on Day 2 after IS Onset

**Fig. (4):**
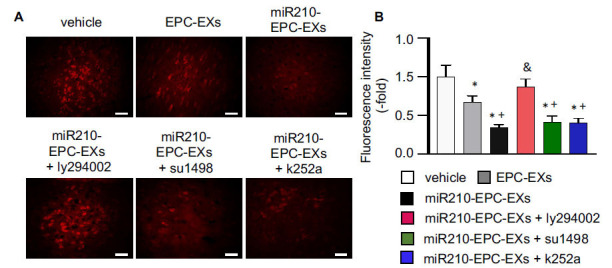
miR-210-EPC-EXs effectively reduce ROS overproduction in the peri-infarct area on day 2 following MCAO. (**A**) Representative images showing the ROS signals revealed by red fluorescence in the cerebral tissue. Scale bars: 40 um; (**B**) Summarized data for ROS. *p < 0.05, *vs.* vehicle; ^+^*p* < 0.05, *vs.* EPC-EXs; ^&^*p* < 0.05, *vs.* miR210-EPC-EXs. n = 8 per group.

Elevated intracellular ROS levels are one of the most important factors for cell injury. To evaluate whether miR210-EPC-EXs have potential effects on oxidative stress, we analyzed the ROS level in the peri-infarct area on day 2 after the treatment. We found that (Fig. **4**) ROS overproduction was significantly lower than that in mice treated by EPC-EXs (*p* < 0.05, *vs.* vehicle). The miR210-EPC-EXs treatment had a better effect on reducing ROS overproduction in the ipsilateral brain than EPC-EXs had (*p* < 0.05, *vs.* EPC-EXs). Such anti-oxidative effect was blocked by the PI3k inhibitor, ly294002 (*p* < 0.05, *vs.* miR210-EPC-EXs), and was partially diminished by the VEGFR2 or TrkB inhibitors, su1498 and k252a (*p* < 0.05, *vs.* vehicle or EPC-EXs).

### 3.5 The Ratios of p-PI3k/PI3k, p-VEGFR2/VEGFRs, and p-TrkB/TrkB were Up-regulated by miR210-EPC-EXs in the Peri-infarct Area of C57BL/6 J Mice on Day 2 after IS Onset

**Fig. (5):**
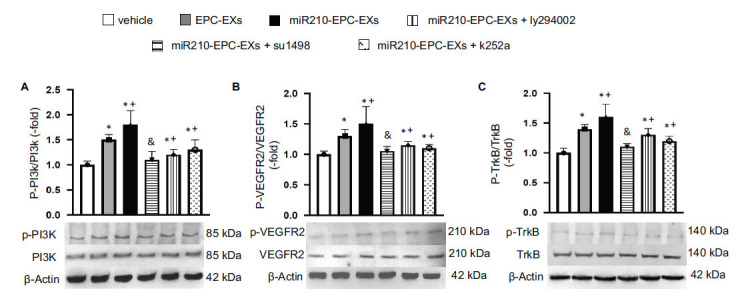
The ratios of p-PI3k/PI3k, p-VEGFR2/VEGFR2, and p-TrkB/TrkB in the ipsilateral brain tissue on day 2 following MCAO. (**A**-**C**) Representative bands, and summarized data showing the expressions of p-PI3K/PI3k, p-VEGFR2/VEGFR2, and p-TrkB/TrkB in different treatment groups. *p < 0.05, *vs.* vehicle; ^+^*p* < 0.05, *vs.* EPC-EXs; ^&^*p* < 0.05, *vs.* miR210-EPC-EXs. n = 8 per group.

The protein expression of PI3k, VEGFR2, and TrkB and their phosphorylation phenotypes in the ipsilateral brain tissue were analyzed by Western Blot. The data showed that p-PI3k/PI3k was elevated by EPC-EXs and miR210-EPC-EXs compared to the vehicle group (*p* < 0.05; Fig. **5A**). The latter had even better efficacy in raising the ratio of p-PI3k/PI3k (*p* < 0.05, *vs.* EPC-EXs or vehicle). As expected, ly294002 significantly blocked the phosphorylation of PI3k (*p* < 0.05, *vs.* miR210-EPC-EXs). Meanwhile, we found that miR210-EPC-EXs significantly upregulated the expression ratios of p-VEGFR2/VEGFRs and p-TrkB/TrkB (*p* < 0.05, *vs.* EPC-EXs or vehicle). su1498 exhibited partially block effects on the phosphorylation of VEGFR2 and k252a diminished the expression of p-TrkB induced by miR210-EPC-EXs (*p* < 0.05, *vs.* miR210-EPC-EXs; Figs. **5B**, **C**). These data suggest that the PI3k/VEGFR2 and TrkB pathways are involved in the favorable effects of miR210-EPC-EXs.

## 4 DISCUSSION

In the present study, we investigated the beneficial effects of miR210-EPC-EXs on protecting the brain against ischemia injury by improving neurological deficit function, reducing apoptosis, and alleviating oxidative stress. We also found that the VEGFR2/PI3k and TrkB/PI3k signaling pathways were responsible for the observed favorable effects of miR210-EPC-EXs.

According to the Minimal information for studies of extracellular vesicles 2018 (MISEV 2018), ultracentrifuge and filtration could be used to isolate the EXs. The method we used in the present study, including filtration and serial centrifuge with ultracentrifuge, has been published as an established method to isolate and identify EXs [32, 38]. The size of the isolated EXs is between 100 nm to 200 nm. The specific markers (CD63, CD9, Tsg101, *etc*.) were also confirmed in the isolated EXs [32, 38]. Stem cell-released EXs have been reported to provide beneficial effects in regenerative medicine and neurological disorders [39-41]. Previous studies have shown that mesenchymal stem cells (MSCs) released EXs promote functional recovery and neurovascular plasticity and regulate axon outgrowth after stroke in animals [42, 43]. The potential role of stem cell EXs in the brain “microenvironment” manifests in several aspects, such as the protection of brain cells (neurons and endothelial cells), maintenance of blood-brain barrier (BBB) hemostasis, and tissue repair (angiogenesis and neurogenesis). In the present study, we focus on the EPC-EXs since Ma *et al*. found that EPC-EXs can protect ECs from hypoxia and reoxygenation injury by improving mitochondrial functionality [21]. The protective effects of EPC-EXs could be attributed to their released factors or their carried cargoes, including miRs and proteins [27, 44]. For example, a previous study discovered the contents of EPC-EXs by using next-generation sequencing and found abundant miR-126 [44]. EPC-EXs were found to prevent microvascular dysfunction and improve sepsis outcomes potentially through the delivery of miR-126 [44]. Our previous studies further confirmed the endogenous contents of miR-126 in EPC-EXs, which subsequentially provide beneficial effects on endothelial dysfunction and promote angiogenesis [27]. Taken together, EPC-EXs could be a potential therapeutic target for ischemia injury.

More importantly, EPC-EXs could function as a vehicle to transfer their carried miRs to the brain and exert protective function [10]. The protective effects of EPC-EXs could be attributed to their released factors or their carried cargoes, including miRs and proteins [31, 45-47]. By taking advantage of EPC-EXs in protecting ischemic brain cells and of miR-210 in reducing cell apoptosis and ROS, we constructed miR210-loaded EPC-EXs by transfecting EPCs with miR-210 mimic and studied the enhanced protective effects of infusion of miR-210-EPC-EXs on acute IS. The intravenous injection was used to introduce the miR210-loaded EPC-EXs to the C57BL/6 mice, which is a normal animal model widely used for medical research. Previous studies have shown that intravenously injected EXs could be tracked in the liver, lung, spleen, and kidney [48, 49]. Our previous study has demonstrated that the injected EXs could cross BBB and merge with the brain ECs, neurons, and astrocytes [10]. To illustrate whether the profound effects of miR210-EPC-EXs are related to their carried endogenous miR-126 and exogenously loaded miR-210, we measured the miR-210 and other EPC exosomal miRs, such as miR-126 levels in the ipsilateral brain. The results showed that the miR-210 level was remarkably up-regulated in the brain tissue of mice that received miR210-EPC-EXs infusion. Meanwhile, the miR-126 level was also elevated in both treatment groups, which is consistent with the findings in diabetic stroke mice showing that EPC-EXs infusion can raise miR-126 expression in the brain tissue [10]. Together these data suggest that miR210-EPC-EXs infusion can convey miR-210 and miR-126 into the brain cells.

For the functional outcome evaluation, we found that EPC-EXs could reduce cerebral infarct volume and improve sensorimotor function. These protective effects are similar to the effects that MSC-EXs provided in the previous studies [42, 43]. Moreover, we found that miR210-EPC-EXs exhibited a higher efficiency than EPC-EXs. Meanwhile, we observed that the cell apoptosis and oxidative stress significantly decreased in the peri-infarct area of miR210-EPC-EXs treated mice than in mice receiving EPC-EXs treatment. These findings indicate that the therapeutic effects of miR210-EPC-EXs in protecting the brain against ischemia injury in the acute phase were better than EPC-EXs. This agrees with previous reports showing that miR-210 could enhance the favorable effects of progenitor cell exosomes in protecting cells against hypoxia or hypertension risk factor-induced injury [21, 23].

To further reveal the downstream signals related to miR-210 and miR-126, we first measured the levels of ligands such as VEGF and BDNF, two important neurotrophins for brain tissue function. We found that both VEGF and BDNF expressions were elevated in mice ipsilateral brains receiving miR210-EPC-EXs or EPC-EXs infusion. As revealed by previous studies, the VEGF/VEGFR2 and BDNF/TrkB signaling pathways participate in regulating a wide variety of brain cell functions, such as cell survival and neurovascular regeneration [50], the outgrowth of axons and dendrites, synaptogenesis, *etc* [51]. Given the raised levels of VEGF and BDNF, the underlying mechanisms for the protective effects of miR210-EPC-EXs could be related to VEGF/VEGFR2 and BDNF/TrkB signaling pathways. To test this hypothesis, we applied the PI3k and relative VEGFR2 and TrkB pathway inhibitors in some experimental mice before treatment. We found that the PI3k inhibitor ly294002 completely blocked the effects of miR210-EPC-EXs on the acute stroke brain as indicated by enlarged infarct volume, a higher rate of Tunnel+ cells, and ROS overproduction in the peri-infarct area. Meanwhile, the phosphorylation of PI3k has significantly reduced in mice that received ly294002 and miR210-EPC-EXs. This data suggests that the PI3k signal is a significant signaling pathway for miR210-EPC-EXs. In addition, our data revealed that the phosphorylation of VEGFR2 and TrkB was significantly decreased in mice that received miR210-EPC-EXs combined with VEGFR2 inhibitor su1498 or TrkB inhibitor su252a, indicating that miR-210 loading can enhance the protective effects of EPC-EXs through the VEGFR2/PI3k and TrkB/PI3k pathways.

## CONCLUSION AND LIMITATIONS

In summary, our study demonstrates that the infusion of miR210-EPC-EXs exhibited enhanced therapeutic effects in protecting the brain against acute ischemic stroke by reducing infarct volume and cell apoptosis and alleviating oxidative stress, thereby improving the neurological deficit function. Such effects might be ascribed to the exosomal miR-210, and miR-126 activated VEGFR2/PI3k and TrkB/PI3k signal pathways. However, more studies are needed to address direct upstream or downstream relationships between miR210-EPC-EXs and the pathway inhibitors since these inhibitors can also exert direct effects on hypoxia-related injuries. In addition, the effects of EPC-EX-miR210 on long-term functional recovery in ischemic stroke are under investigation, which will provide more evidence to support the protective effects of EPC-EX-miR210 in brain ischemia injury.

## AUTHORS’ CONTRIBUTIONS

J.B. designed and supervised the research and interpreted the data; J.W., S.C., and H.S. performed the study, collected and analyzed data, and made the figures; J.W. and J.B. performed the statistical analysis and interpreted the data; J.B. and J.W. drafted the manuscript; J. B. and Y.C. revised and approved the manuscript.

## Figures and Tables

**Fig. (2):**
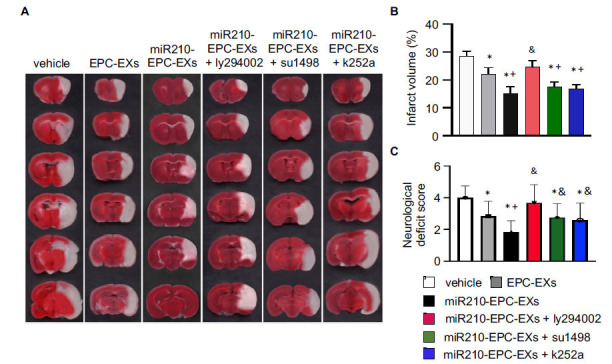
miR-210-EPC-EXs are more effective in reducing infarct volume on day 2 following MCAO. (**A**) Representative TTC staining images show the infarcted brain tissue (white area) in different treatment groups; (**B**) Summarized data of the infarct volume; n = 8 per group. (**C**) Summarized data of the NDS in different treatment groups; n = 12 per group. *p < 0.05, *vs.* vehicle; ^+^*p* < 0.05, *vs.* EPC-EXs; ^&^*p* < 0.05, *vs.* miR210-EPC-EXs.

**Fig. (3):**
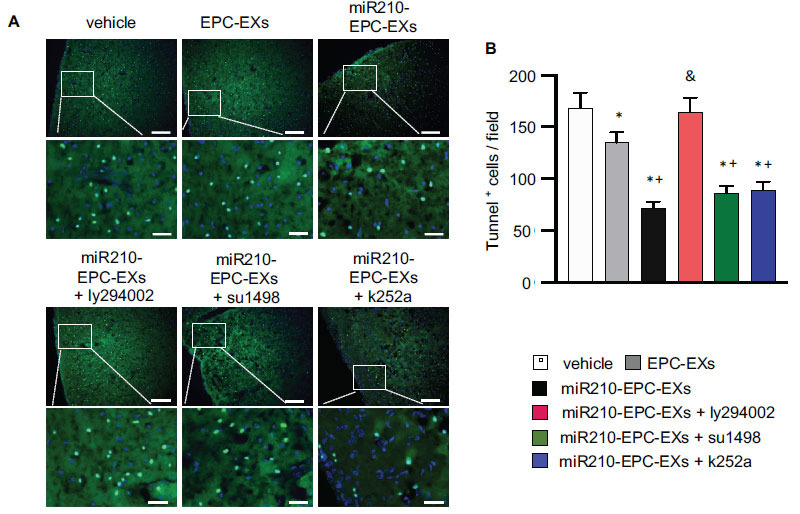
miR-210-EPC-EXs is more effective in alleviating cell apoptosis in the peri-infarct brain tissue on day 2 following MCAO. (**A**) Representative images showing the Tunnel + cells in the cerebral tissue. Green: Tunnel+ cells; Blue: DAPI; Scale bars: 100 um for the upper panel and 25 um for the bottom panel. (**B**) Summarized data for the Tunnel+ cells in each field. *p < 0.05, *vs.* vehicle; ^+^*p* < 0.05, *vs.* EPC-EXs; ^&^*p* < 0.05, *vs.* miR210-EPC-EXs. n = 8 per group.

## Data Availability

The datasets used and/or analyzed during the current study with the corresponding author and will be provided upon reasonable request.

## LIST OF ABBREVIATIONS

BBB: Blood-Brain Barrier
BDNF: Brain-Derived Neurotrophic Factor
DAPI: 4’, 6-Diamidino-2-Phenylindole
DHE: Dihydroethidium
ECs: Endothelial Cells
EPC-EXs: Endothelial Progenitor Cell-Released Exosomes
EPCs: Endothelial Progenitor Cells
EXs: Exosomes
IS: Ischemic Stroke
MCAO: Middle Cerebral Artery Occlusion
miR: microRNA
miR210-EPC-EXs: miR-210-Loaded EPC-EXs
MSCs: Mesenchymal Stem Cells
NDS: Neurological Deficit Score
NTA: Nanotracking Analysis
PBS: Phosphate Buffer Saline
PFA: Paraformaldehyde
PI3K: PI3 Kinase
ROS: Reactive Oxygen Species
TdT: Terminal Deoxynucleotidyl Transferase
TrkB: Tyrosine Receptor Kinase B
TTC: 2,3,5-Triphenyltetrazolium Chloride
TUNEL: dUTP Nick-End Labeling
VEGF-A: Vascular Endothelial Growth Factor A
